# Rac1 Selective Activation Improves Retina Ganglion Cell Survival and Regeneration

**DOI:** 10.1371/journal.pone.0064350

**Published:** 2013-05-29

**Authors:** Erika Lorenzetto, Michele Ettorre, Valeria Pontelli, Matteo Bolomini-Vittori, Silvia Bolognin, Simone Zorzan, Carlo Laudanna, Mario Buffelli

**Affiliations:** 1 Department of Neurological, Neuropsychological, Morphological and Motor Sciences, Section of Physiology, University of Verona, Verona, Italy; 2 Department of Pathology, Section of General Pathology, University of Verona, Verona, Italy; 3 Center for Biomedical Computing, University of Verona, Verona, Italy; 4 National Institute of Neuroscience, Verona, Italy; Massachusetts Eye & Ear Infirmary, Harvard Medical School, United States of America

## Abstract

In adult mammals, after optic nerve injury, retinal ganglion cells (RGCs) do not regenerate their axons and most of them die by apoptosis within a few days. Recently, several strategies that activate neuronal intracellular pathways were proposed to prevent such degenerative processes. The rho-related small GTPase Rac1 is part of a complex, still not fully understood, intracellular signaling network, mediating in neurons many effects, including axon growth and cell survival. However, its role in neuronal survival and regeneration *in vivo* has not yet been properly investigated. To address this point we intravitreally injected selective cell-penetrating Rac1 mutants after optic nerve crush and studied the effect on RGC survival and axonal regeneration. We injected two well-characterized L61 constitutively active Tat-Rac1 fusion protein mutants, in which a second F37A or Y40C mutation confers selectivity in downstream signaling pathways. Results showed that, 15 days after crush, both mutants were able to improve survival and to prevent dendrite degeneration, while the one harboring the F37A mutation also improved axonal regeneration. The treatment with F37A mutant for one month did not improve the axonal elongation respect to 15 days. Furthermore, we found an increase of Pak1 T212 phosphorylation and ERK1/2 expression in RGCs after F37A treatment, whereas ERK1/2 was more activated in glial cells after Y40C administration. Our data suggest that the selective activation of distinct Rac1-dependent pathways could represent a therapeutic strategy to counteract neuronal degenerative processes in the retina.

## Introduction

In adult mammalian central nervous system (CNS) neurons fail to regenerate after injury. Many approaches have been tried with the aim of promoting neuronal survival and reactivation of an axonal growth program that is normally shut down at the end of development [1], [2]. Although the intracellular signaling underlying neuronal survival and axonal regeneration are still partially understood, increasing evidence has shown that stimulation of intrinsic pathways could be an effective strategy [2]–[5].

Rac1 is a Rho-related small GTPase that regulates cytoskeletal dynamics and plays a critical role in neuronal development, axon growth and cell survival [6]. The constitutively active (CA) form of Rac1 was able to promote axonal growth through an inhibitory environment both *in vitro* [7] and *in vivo* [8] and to promote survival *in vitro* [9], [10]. However, Kusano and coworkers obtained regeneration by using a dominant negative (DN) Rac1 on a model of sciatic nerve injury [11]. Indeed, a critical point is that Rac1 is a signaling network hub and can potentially interact with several downstream effectors [12]; hence, its pure CA and DN forms could influence simultaneously many downstream, and likely concurrent, signaling pathways, leading to contrasting effects.

In order to investigate the role of Rac1 in neuronal survival and regeneration *in vivo* and to limit the inherent uncertainty due to the multitude of possible interactions, we coupled the pure constitutively activating L61 mutation to a second mutation in the effector domain (F37A or Y40C) in order to block the specific interaction of Rac1 with a pool of downstream effectors. It was shown that after F37A point mutation Rac1 retains the ability to interact with PAK and to influence JNK activation, whereas for the Y40C mutant the above-mentioned interactions are blocked, at least *in vitro* [13], [14]. We fused the mutants with a Tat Trojan sequence to mediate the cell internalization. We used the well known model of optic nerve injury to study the role of Rac1 in neuronal survival and regeneration, injecting intravitreally (ivit) the two double mutants after crush. These mutants have been previously characterized [13]–[15], but to our knowledge, they have never been used *in vivo* in the CNS.

Our results showed that selective activation of Rac1-dependent signaling pathways improved cell survival and axonal regeneration until 15 days post-injury. Moreover, after Rac1L61F37A treatment we observed an increase of p21-activated kinase 1 (Pak1) T212 phosphorylation in RGCs together with extracellular signal-regulated kinase 1 and 2 (ERK1/2) upregulation. On the other hand the L61Y40C increased ERK1/2 activation in radial glia. Our study clarifies the role of Rac1 as a pro-survival signaling molecule in neurons *in vivo* and supports the possibility of using the Trojan peptide-based approach in the treatment of neuronal damages.

## Methods

### Animals

Mice were maintained under standard environmental conditions (temperature, humidity, 12 h/12 h light/dark cycle, with water and food *ad libitum*) under veterinarian assistance. Animals handling and surgery were performed following a protocol which received approval by the Animal Care and Use Committee of the University of Verona (CIRSAL), and authorization by the Italian Ministry of Health, in strict adherence to the European Communities Council directives (86/609/EEC), minimizing the number of animals used and avoiding their suffering.

The 2 transgenic models used were purchased from the Jackson laboratory (Bar Harbor, ME, USA): B6.Cg-Tg(Thy1- YFPH)2Jrs/2J (referred to as YFPH) and B6.CBA-Tg(Thy1-Brainbow1.0)LLich/J (referred to as Brainbow 1.0 ) mice. YFPH mice were genotyped by PCR. Some immunofluorescence experiments were performed on C57BL6/J mice to avoid the presence of endogenous fluorochromes. We used the YFPH mouse line, in which a few RGCs constitutively express the yellow fluorescent protein (YFP) under the *thy1* promoter [16], to evaluate the survival and the whole cell morphology of RGCs. Since in YFPH mice the number of fluorescent axons in the optic nerve is very small and variable among mice, we used the Brainbow mice to assess regeneration [17]. In these mice the YFP is expressed only after Cre-dependent recombination that we obtained by an Adeno-Associated Virus carrying the green fluorescent protein-fused Cre recombinase (AAV-Cre-GFP). If we perform the AAV-Cre-GFP injection around the day of crush, we will achieve recombination and expression of YFP only in the axons of surviving neurons (for more details see Fig. S1).

### Tat-protein Fusion Production

The penetration of mutant proteins in cells was achieved by a Tat Trojan sequence, an approach that has been previously used [18]. We constructed a series of specific Tat fusion protein expression vectors based on pRSETa-b-c vectors (Life Technologies, Carlsbad, CA, USA), designed for high-level protein expression and purification on nickel columns upon expression in Escherichia coli. A detailed description of Trojan peptides production is available in the supplementary material of [15]. A scheme of the recombinant protein mutants with all the mutations and a complete amino acid sequence are available in Fig. S2.

### Optic Nerve Crush and Intravitreal Injection Procedure

**Figure 1:**
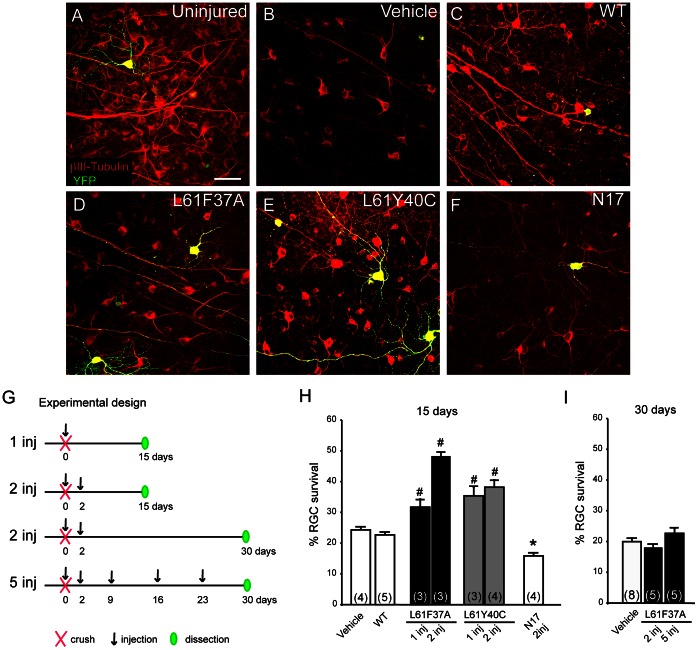
Rac1 selective activation increased RGC survival until 15 days after optic nerve crush in YFPH mice. Representative confocal images of flat mounted normal (A), and injured retinas after treatments with 2 injections (at day 0 and day 2 post lesion) of either vehicle (B) or Rac1WT (C), or Rac1L61F37A (D), or Rac1L61Y40C (E), or the DN Rac1-N17 (F). Retinas were excised at 15dpl and stained in order to detect RGCs (βIII tubulin, red), and YFP (anti-GFP, green). The dramatic neuronal loss observed in the vehicle-treated mice (B) was reduced in mice treated with either Rac1L61F37A (D) or Rac1L61Y40C (E). Scale bar 50 µm. Mutants were tested for different experimental protocols, whose scheme is shown in (G). YFPH mouse optic nerves were crushed at day 0 and, after one or more injections of Rac1 mutants, WT or vehicle, retinas were dissected 15 or 30 days post crush in order to investigate RGCs survival. After staining for βIII tubulin, flat mounted retinas were imaged and processed for the evaluation of survival as described in methods. (H-I**)** Quantification of RGC survival after optic nerve crush and Rac1 selective activation at 15 and 30 days post lesion in YFPH mice. The results at 15 dpl are shown in H, where both CA mutants were able to improve survival. Only the L61F37A exerted a dose dependent effect, whereas the DN N17 mutant decreased survival. At 30 dpl (I) the survival of Rac1 L61F37A treated mice decreased at the level of controls, even after repetitive injections, indicating that the mutant was unable to sustain survival at 30 days. Data are mean ±SEM, N is in brackets. *p<0,05; ^#^p<0.01 versus the controls (one way ANOVA followed by LSD post hoc test).

Animals were anesthetized with trimbromethanol (TBE, ip dose of 0,4 g/kg body weight) and the left optic nerve was exposed intraorbitally. The onset of RGC death depends on the distance of the injury from the retina [19], so we performed an intraorbital lesion of optic nerve at a fixed distance of 2 mm from the eye, applying a pressure with jewelers’ forceps for 10 s [20], [21]. The eyes of experimental and control animals were ivit injected with mutants or vehicle (PBS): each injection consisted of 1 µl at 5 µM concentration of Tat-protein. Injections were performed by using a heat-pulled glass capillary connected to a picospritzer (Parker Instrumentation, NH, USA), (0.05 ms pulse duration, pressure of 10 psi). Injections were given slowly and the capillary was maintained in position for additional 10 min to minimize protein loss through the injection tract. The first injection was done just after optic nerve crush, and then animals were allowed to recover in their cage. We performed one or more injections in the following days according to different schemes of treatment described in the Fig. 1G. At the end of the experiment, 15 or 30 days post crush, mice were sacrificed by terminal anesthesia and transcardially perfused with 4% paraformaldehyde in PBS, pH 7.4. The eyes and optic nerves were removed, postfixed and processed for immunofluorescence as described below or directly mounted and studied by using a TCS-SP5 confocal microscope (Leica-Mycrosystems, Wetzlar, Germany).

In the analysis of regeneration (15 and 30 days post crush), Brainbow 1.0 mice were also ivit injected with 1 µl of AAV-Cre-GFP two days before the optic nerve crush in order to allow the recombination and the expression of YFP in the axons.

At the dissection step, eyes showing lens injury were excluded from the study, because survival and regeneration of RGCs is stimulated by crystal lens damage [20]. Since inflammation could affect survival and regeneration [22], all controls received the same number of vehicle injections of the experimental mice.

### Immunofluorescence

Retinas (whole tissue or 15 µm thick cryosections, depending on the experiment) were permeabilized with the following solution: 2% Bovine Serum Albumin, 2% Normal Goat Serum, 0,3% of Triton X-100, in PBS, pH 7.4 for 30 minutes. Then tissues were incubated 24 h at 4°C with primary antibodies and, after washes, with the proper secondary Alexa Fluor-conjugated antibodies (Life Technologies, Carlsbad, CA, USA) for 1 h at RT. Nuclei were stained by DAPI (Sigma-Aldrich, St Louis, MO, USA). After mounting, retinas were studied by confocal microscopy.

The following primary antibodies were used: anti-βIII tubulin, anti-Pak1, anti Phospho-Pak1 (Thr 212), anti-JNK and anti-di Phospho-JNK (Thr 183 and Tyr 185) were purchased from Sigma-Aldrich (St Louis, MO, USA), Alexa 488-conjugated anti-GFP and anti-Histag were purchased from Life Technologies (Carlsbad, CA, USA), anti-ERK1/2 and anti-Phospho-ERK1/2 (Thr 202 and Tyr 204) were purchased from Cell Signaling, (Beverly, MA, USA), anti-GFAP (MAB360) was purchased from Millipore (Billerica, MA, USA).

### Confocal Imaging Analysis

#### Analysis of cell survival

Six confocal stacks of the RGC layer were acquired from each flat mounted retina after βIII tubulin staining at 400× magnification (40× oil immersion objective, HCX PL APO Lambda blue,1.25 numerical aperture (NA), Leica Microsystems, Wetzlar, Germany). βIII tubulin was used as a marker for surviving RGCs as it is an efficient and reliable method for selective labeling of viable RGCs [23]. Given that the neuronal density changes with the distance from the optic disc, we acquired images at two different distances: 3 images from the medial and 3 from the peripheral retina. A manual count of RGCs was done on the z-stack. Since the crush was performed mono-laterally, the mean RGC density of the contralateral eyes was used as reference for the number of RGCs before crush. The ratio, expressed as percentage, between the RGC density of the crushed retina and the reference was referred to as the survival rate.

#### Analysis of dendrite morphology

YFPH positive neurons (10 to 20 neurons per treatment) were acquired by confocal microscopy at 400× magnification by using the 40× oil immersion objective (1.25 NA). In the YFPH retina RGCs are well separated between each other. Given the presence of 6 different morphological types of RGCs in the mouse retina, we focused only on type 4 neurons that are the most resistant to death after crush [24]. Type 4 neurons were recognized on the basis of their distance from the optic disc (1.8–2 mm). Z-stacks were acquired and the maximum intensity projections (MIPs) were obtained by using the LAS-AF software (Leica-Microsystems, Wetzlar, Germany). On the MIPs the Sholl analysis was performed by using the ImageJ specific plugin (NIH, Bethesda, MD, USA). In this analysis, after application of a treshold, a series of concentric circles with increasing radii were automatically drawn by the software starting from the center of the cell body that was manually set (5 µm radius step size; starting radius 10 µm). The program evaluates the number of dendritic intersections at each value of the radius, resulting in a main plot (N of intersections against the distance form soma), together with a series of parameters that we used for the statistical analysis: the maximum number of intersections, the ramification index and the critical value.

#### Analysis of regeneration

Brainbow1.0 whole mounted nerves were acquired at 400× magnification (40× oil immersion objective, 1.25 NA) by laser scanning microscopy. We acquired adjacent z-stacks (100 µm of z-thickness) and after mosaic merge and identification of the crush site, regenerating axons were manually counted in the images every 100 µm starting from the crush site. The number of axons was plotted against the distal nerve length.

#### Analysis of Pak/ERK/JNK signal intensities

After staining the retina cryosections, 3 images per retina were randomly acquired from 3 to 6 mice per treatment at 400× magnification (40× oil immersion objective, 1.25 NA). In order to compare the fluorescence intensities coming from different samples, we performed the staining with the same antibody solutions, on the same day and we kept constant all the acquisition parameters during the imaging sessions. On the z-stack a ROI was drawn in correspondence of the ganglion cell layer and the average intensity was measured over the entire layer by using the LAS-AF software. Data were expressed as normalized data on the control value (e. g. the normal retina).

All images were processed by using the LAS-AF software (Leica-Mycrosystems, Wetzlar, Germany) and ImageJ (NIH, Bethesda, MD, USA).

### Statistical Analysis

For the analysis of cell survival and dendrite intersections (Sholl analysis) data were evaluated by ANOVA followed by LSD post hoc test. For the evaluation of axonal regeneration we counted the number of axons at different distances from the crush site from 100 to 1000 µm (100 µm bin size). Data were processed for repeated measure ANOVA followed by LSD post-hoc test, using the length as within subjects factor and the treatments as between subjects factor. For the evaluation of differences in the number of regenerating axons at each length (for instance only at 100 µm) we used the one way ANOVA followed by LSD post hoc test.

The evaluation of immunofluorescence intensity was done by ANOVA followed by LSD post-hoc test.

All statistical analyses were performed by SPSS (IBM, New York, NY, USA) software. An alpha level of 0.05 was considered as significant.

## Results

### Rac1 Selective Activation Promoted RGC Survival 15 Days after Optic Nerve Crush

To unveil the role of Rac1 on neuronal survival *in vivo* we used YFPH mouse line in which a few RGCs constitutively express YFP [16]. We performed an optic nerve crush and injected ivit two CA-Tat-Rac1 selective double mutants: the L61F37A and the L61Y40C. As controls, we used vehicle and a Tat-Rac1WT isoform that can freely cycle between the active and the inactive state responding to the cell activity. Moreover some mice were injected with the Tat-Rac1N17 that is the DN Rac1 isoform. All the experimental and the control treated mice received the same number of injections and eyes with signs of crystal lens damage were excluded from this study (see methods).

In preliminary experiments, we tested the effect of different doses of the Tat-Rac1 mutants on RGC viability in a concentration range between 5 to 80 µM. We found that 5 to 10 µM gave the highest RGC viability (data not shown) and thus we decided to use 5 µM in this study. Fig. 1 shows examples of normal (A) and crushed retinas (B to F), with or without Rac1 mutant treatment: eyes were injected twice and excised 15 days post-lesion as shown in the scheme of Fig. 1G (second treatment: 2 inj 15 days). A relevant neuronal loss was found in controls (B and C), while the treatment with either the Rac1 L61F37A (D) or the L61Y40C (E) protected RGCs from death. On the contrary the DN isoform exacerbated neuronal death.

Fig. 1H and I show the quantification of RCG survival in mice treated according to the different protocols illustrated in G: i) a single injection performed the day of the crush (day 0) ii) the double injections performed at day 0 and day 2 with excision at 15 or iii) at 30 days and iv) the repetitive injection over 30 days.

In controls the 24% of neurons survived at 15 days post lesion (dpl) but in mice treated with Rac1L61F37A double injection the percentage increased to 50%. The L61F37A was the most effective mutant in a dose-dependent fashion. The L61Y40C mutant was also able to increase survival but at a lower level and without apparent dose-dependency. On the contrary the N17 mutant significantly impaired the neuronal survival. Given these results only the F37A mutant was tested for longer time.

When we look at the plot 1I relative to the two treatments at 30 dpl, we observed that the L61F37A was unable to sustain RGC survival. Since vehicle treatment resulted in the same survival after 2 or 5 injections, we grouped together the data showing a single column for controls (n = 8).

These data suggest that Rac1 selective activation is able to improve RGC survival until 15 dpl.

**Figure 2:**
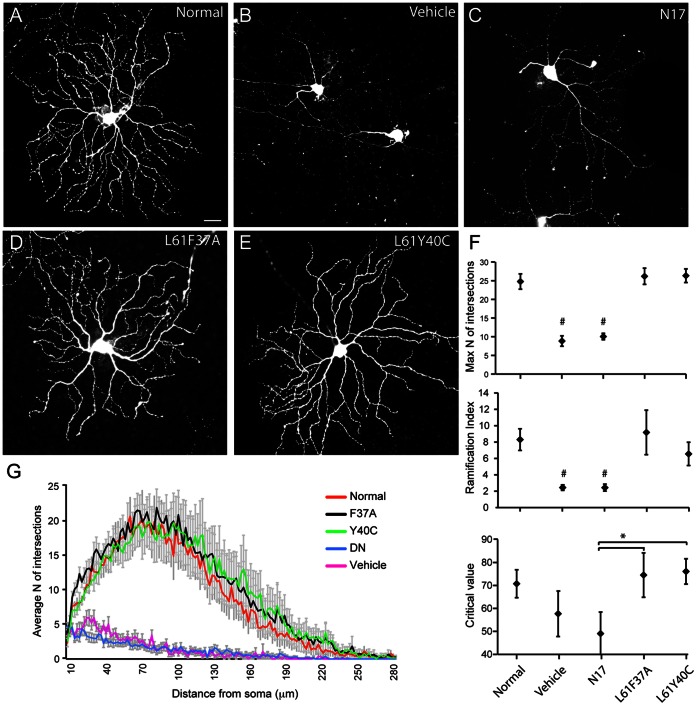
Rac1 selective activation prevents the dendrite atrophy occurring after crush. Representative confocal maximum projections of YFPH mouse RGCs 15 days after crush and double injection of either vehicle or CA or DN mutants. A normal RGC is shown for comparison (A). After Rac1L61F37A and Y40C treatment (D and E respectively) the dendritic atrophy is prevented, whereas the same extent of degeneration was found in control (B) and after DN treatment (C). The plots relative to the Sholl analysis for the different treatments are shown in F–G: (F) the maximum number of intersections, the ramification index and the critical value were used for statistical evaluation (10 to 20 neurons per treatment), whereas (G) the N of intersections against the distance from the soma shows the morphological changes along the whole dendritic tree. Scale bar 20 µm. # p<0,01 versus the control, the L61F37A and the L61Y40C; *p<0,05 versus the indicated group (one way ANOVA followed by LSD post hoc test).

The retinas receiving double injections and excised at 15 dpl were processed for the evaluation of dendrite morphology. Fig. 2 shows representative confocal images of class 4 RGCs (Leung et al., 2011) from the different treatments. The morphology of neurons was well conserved after Rac1 selective activation (D-E), while in controls and in N17 treatment it was dramatically altered (B and C). Results indicate that Rac1 selective activation was able to prevent the dendrite atrophy naturally occurring after crush. The Sholl analysis confirmed and supported the differences observed, revealing a dramatic decrease of complexity of the dendritic tree after vehicle and DN treatments as compared to Rac1 selective activation (Fig. 2 F–G). Moreover, since we observed opposite effects for CA and DN Rac1 isoforms, we can exclude that the differences on survival and dendritic trophism are related to the blocking of the GTPase cycling.

### Selective Activation of Rac1 Promoted RGCs Axonal Regeneration Following Crush Injury

**Figure 3:**
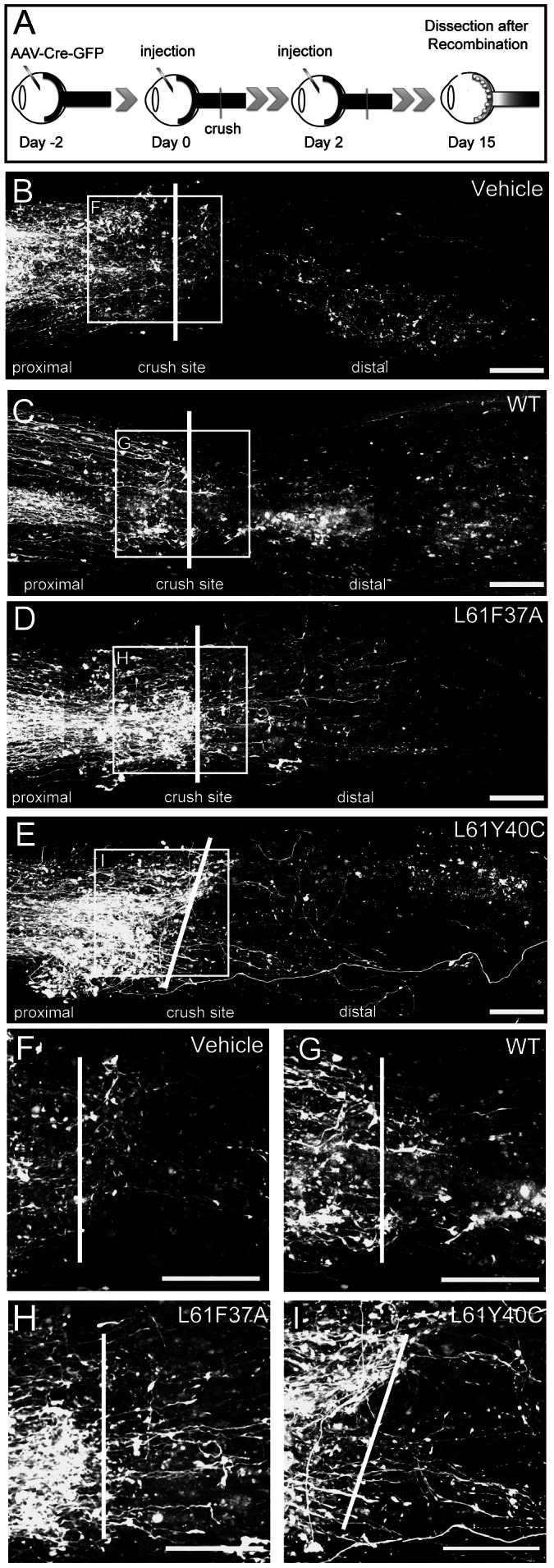
Rac1 selective activation promotes axonal regeneration after optic nerve crush in Brainbow mice. In Brainbow mice the injection of the AAV-Cre-GFP around the day of crush triggers a genetic recombination that leads to YFP expression (white false color) only in surviving neurons. We injected either the Tat-Rac1 mutants, WT or vehicle on the day of crush (day 0) and on day 2 and studied regeneration 15 days post lesion. A scheme of the treatment is in A. Nerves were acquired and studied by confocal microscopy. Single examples of whole mounted nerves after mosaic merge reconstruction are given in B to E, and are relative to vehicle (B), Rac1WT (C), L61F37A mutant (D) and L61Y40C mutant (E) double injections. The crush sites of B, C, D and E are enlarged in F, G, H and I respectively. By comparison of the panels it is clear that after treatment with L61F37A a higher number of axons is able to cross the crush site and run distally (D and H). Scale bars 100 µm.

In order to study axonal regeneration, we used the Brainbow 1.0 mouse [17], in which the red fluorescent protein (RFP) is basally expressed by all neurons under the *thy1* promoter. In this transgenic line the ivit injection, around the day of crush, of an AAV-Cre-GFP triggers the expression of yellow and cyan fluorescent proteins (YFP and CFP) only in the surviving neurons (see methods and Fig. S1). The RFP gene is concomitantly deleted in recombining neurons. After the recombination the CFP signal in the optic nerve was faint respect to the YFP, thus we excluded it from the analysis and showed only the YFP signal in white false color (Fig. 3). The recombination occurs in about the 75% of neurons, so in this model the regeneration is underestimated.

**Figure 4:**
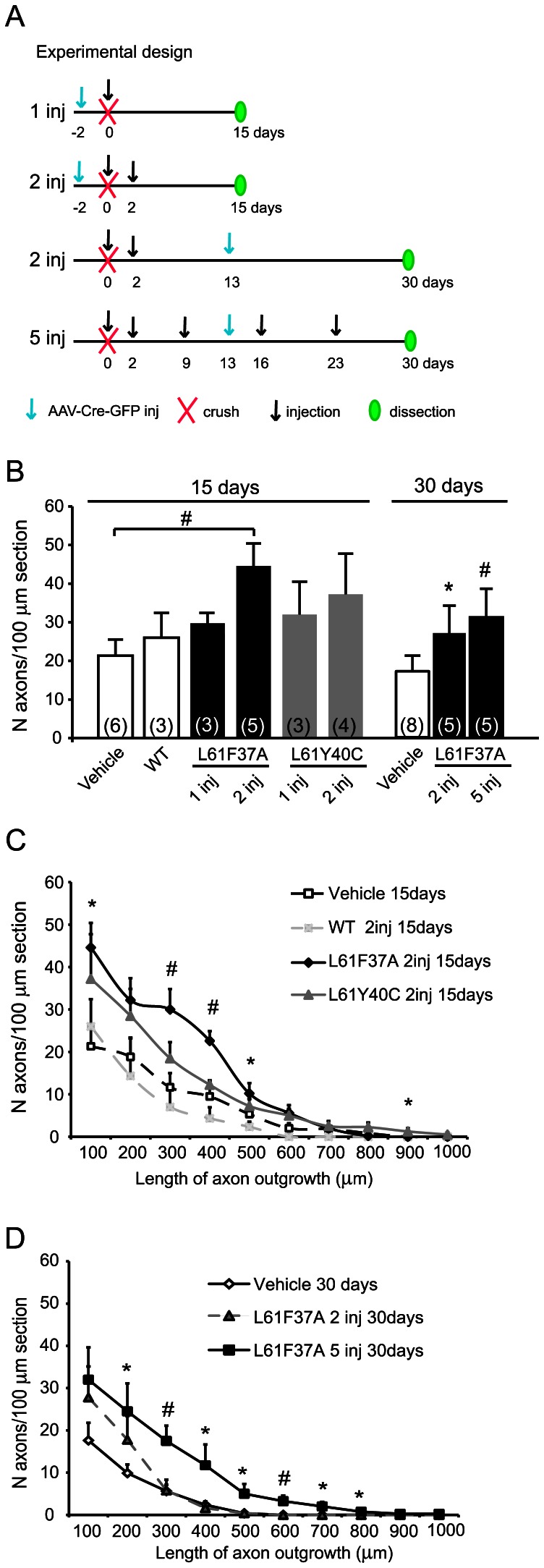
Quantification of axonal regeneration after optic nerve crush and Rac1 selective activation in Brainbow mice. Brainbow mouse optic nerves were crushed at day 0 and, after one or more injections of either Rac1 mutants, WT or vehicle, were dissected at 15 or 30 days post crush in order to investigate regeneration. A scheme of the different treatments is given in A. Nerves were studied by confocal microscopy and the results of the regeneration study are plotted in B, C and D. Only the double injection of L61F37A was able to increase the average number of axons crossing the crush site per 100 µm of nerve z-section (B) at 15 days post lesion. The number of regenerating axons is higher than control also after 2 and 5 injections of L61F37A at 30 dpl. The data at 15 days are confirmed also by the analysis of length distribution in the entire distal stump (C). The same analysis at 30 days (D) revealed that, despite after 2 and 5 L61F37A injections the total number of regenerating neurons are similar, the repetitive treatment resulted in longer axons. Since we found no differences between the various vehicle injection protocols of treatment at 15 and 30 days, we put together the data of the controls on the same column/curve (n = 6 and 8). Data are mean ±SEM. N is in brackets. ^#^p<0.01, *p<0.05 by ANOVA (LSD post hoc test).

To test whether Rac1 selective activation could improve axonal regeneration, we injected the virus (day −2) and after 2 days we performed the crush (day 0) followed by ivit injection of the two different Tat-Rac1 mutants, with the same protocols of treatment used for the survival study (see also scheme in Fig. 4A). First we evaluated the regeneration 15 days post lesion. Fig. 3 shows examples of confocal images of mice treated with two injections and excised at 15 dpl (see scheme 3A or 4A second treatment). Here a higher number of regenerating axons spanning the crush site were found in L61F37A treated animals (Fig. 3 D and H) respect to controls (B, C, F and G). After quantification, we found that double injection of L61F37A significantly increased the average number of regenerating axons as compared to controls at 15 dpl (Fig. 4B). This data was also confirmed by the analysis of the length distribution in the distal nerve stump (Fig. 4C). Conversely, neither the single L61F37A nor the L61Y40C single and double injections showed any significant effects.

**Figure 5:**
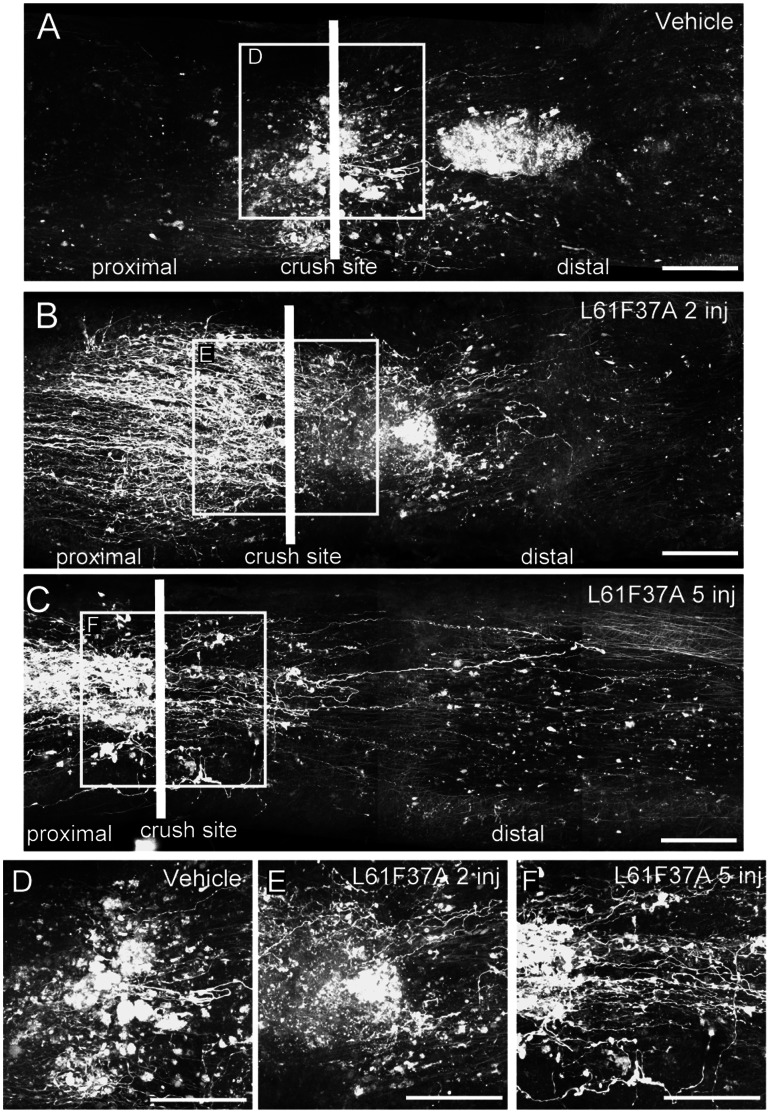
Long-term effects on axonal regrowth of Rac1L61F37A treatment in Brainbow mice. Single examples of whole mounted Brainbow mouse nerves after mosaic merge reconstruction, relative to the 30 dpl treatments described in figure 4A and D. Images shows treatments with vehicle (A), 2 (B) and 5 (C) L61F37A injections. The crush sites of A, B and C are enlarged in D, E and F respectively. Longer axons were found distally from the crush site after multiple injections of the Rac1 mutant. Scale bars 100 µm.

In order to define the effect of long-term treatments of L61F37A on axonal regeneration, we performed experiments in which nerves were excised 30 dpl after 2 (on day 0 and 2) or 5 injections (see scheme in Fig. 4A, 2inj and 5 inj 30 days). As shown in Fig. 5, after 5 injections of L61F37A many axons were found spanning the crush site (Fig. 5 C and F). This was not observed neither after 5 vehicle injections (A, D), nor after double injection of mutant (Fig. 5 B, E).

As shown in the quantification (Fig. 4B 30 days), at 30 dpl more regenerating axons were found after L61F37A treatments respect to their control. Moreover, at the same time point, 5 injections with L61F37A resulted in longer axons with respect to 2 injections (Fig. 4D). This is important if we consider that the survival rate at 30 dpl is the same in controls and in treated animals (Fig. 1I). Since we found no differences between the various vehicle injection protocols of treatment at 15 and 30 dpl, we grouped together the data of the controls (n = 6 for 15 dpl and 8 for 30 dpl).

Interestingly, when we compared data obtained at 15 and 30 dpl, we found no statistical differences between the double L61F37A injection at 15 dpl and the 5 injections at 30 dpl, suggesting that repetitive injections of mutant are unable to improve regeneration after 15 dpl. On the contrary, we found a significant decrease in axonal length between the controls at 15 dpl and those at 30 dpl and also between the double injection of L61F37A at 15 and at 30 dpl (Fig. 4 C *vs* D). It seems likely that sustained delivery of L61F37A could prevent further nerve degeneration.

Our data suggest that Rac1 selective activation may improve regeneration until 15 days post injury, and that the repetitive treatment could be able to delay the axonal degeneration.

### Injection of Rac1 CA Mutants Increased Pak1 Phosphorylation and ERK1/2 Activation in Different Retina Cell Types

In order to study the half-life of the Tat-proteins we performed Histag specific staining (see Fig. S3 and S4) from 2 to 4 days after a single Tat-protein injection. We observed that after 2 and 3 days from the injection the mutant protein was still present in the retina, while the signal was no more detectable at 4 days.

**Figure 6:**
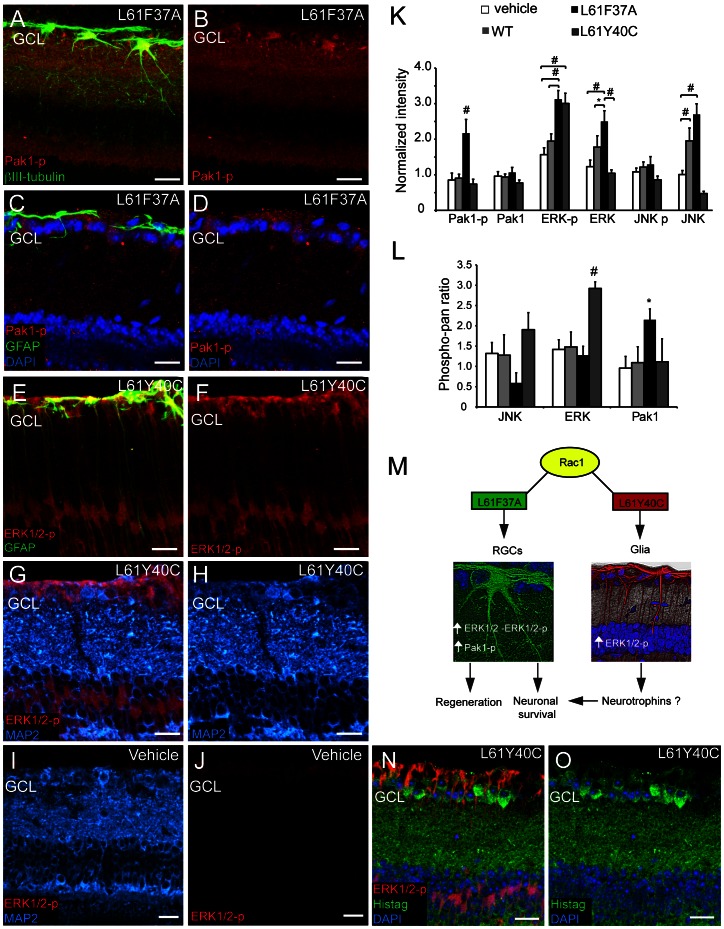
Phosphorylation of Pak1 and upregulation of ERK1/2 after injections of Rac1 mutants. Retina sections were immunostained by antibodies against the pan-specific and the phosphorylated form of Pak1 (T212), ERK1/2 and JNK. Retinas were dissected and immunostained 3 days after optic nerve crush and ivit treatment with either vehicle, or Rac1WT, or L61F37A or L61Y40C. Some representative relevant images are shown in A to J, N and O. (A–B) Colocalization of phospho-Pak1 (Pak1-p, red) and βIII tubulin (green) indicates that the L61F37A resulted in Pak1-p increase in RGCs, confirmed also by the lack of colocalization with GFAP (C–D). (E–F) Colocalization of phospho-ERK (ERK1/2-p, red) and GFAP (green) indicates that the L61Y40C mutant activated ERK1/2 in retinal glial cells. (G–H) The lack of colocalization between MAP2 (blue) and ERK1/2-p after Y40C treatment indicates that this protein is not activated in neurons. A control is also shown (I–J) where ERK1/2-p positivity is very low. (N–O) Histag staining after L61Y40C treatment showed a clear positivity in RGCs, meanwhile the ERK1/2-p positivity pattern was still glial-like. (K) Semi-quantitative expression of total and phosphorylated level of Pak1, ERK1/2, and JNK measured in correspondence of the ganglion cell layer and normalized on the signal of the normal eye (n = 9 to 15 confocal stacks from 3 to 6 animals). (L) Ratio between the normalized intensity of phosphorylated and total forms of Pak1, ERK1/2 and JNK gives an indication about the degree of kinase activity. Scale bar 30 µm. GCL: ganglion cell layer. (M) We hypothesize that L61F37A effect on survival might be related to the increased expression of ERK1/2 and increased Pak1 phosphorylation in neurons. Meanwhile, L61Y40C is most likely boosting survival through the activation of ERK1/2 in astrocytes.

To assess what proteins could be involved in the effect observed after Rac1L61F37A and Y40C treatment on RGC neurons, we stained retina cryosections with different antibodies recognizing either the pan-specific or the phosphorylated form of some of the most common downstream Rac1 effectors: Pak1, ERK1/2 and the c-jun N-terminal kinase (JNK). We performed the staining 3 days after crush and double injection of Tat-conjugated proteins. In this way, we were quite sure to detect the effect in a period when the Tat-proteins were still present and not yet degraded by cell/tissue metabolism. We found an L61F37A-dependent increase of both total and phospho-ERK1/2 and phospho-Pak1 (T212) in the RGC layer (Fig. 6 K and L**)**. These increments were specifically in RGCs as shown by the colocalization with the βIII tubulin (Fig. 6 A–B). We also found an increase of the total ERK1/2 and JNK as compared to vehicle treatment (Fig. 6K and Fig. S5). However, since we observed the same pattern for JNK expression in the WT-treated animal, we concluded that this effect might not be strictly related to the Rac1 constitutive activation.

As concern the Y40C isoform, the observed colocalization with the glial fibrillary acidic protein (GFAP) together with the evaluation of the phospho/pan antibody ratio, indicated that the Y40C mutant treatment resulted in the activation of ERK1/2 specifically in glial cells (Fig. 6 E-F and K-L) and not in neurons (Fig. 6 G-H). Although the Histag staining after both mutant treatments indicates that the Tat-proteins mainly targets RGCs (we showed only the Histag staining after L61Y40C injection in Fig. 6 N-O and Fig. S4 but the pattern for the F37A isoform is the same, data not shown), we found a surprising but specific positivity for the phospho-ERK1/2 in the radial glia.

In order to confirm the specificity of these effects we injected the mutants in normal eyes and 48h after single injection we repeated the staining on the retinas obtaining the same results (see Fig. S6): after Y40C treatment the level of phospho-ERK1/2 was increased in non-neuronal cells, whereas after F37A treatment the positivity for phospho-Pak1 was increased in RGCs.

Our data suggest that different CA isoforms of Rac1 are able to promote Pak1 phosphorylation and ERK1/2 activation *in vivo* on different cell types acting by distinct mechanisms.

## Discussion

In this study, we demonstrated that two cell-penetrating fusion proteins, selectively modulating the signaling activity of the rho small GTPase Rac1, contrasted the RGC death and stimulated the axon regrowth in a model of optic nerve injury *in vivo*. Our data showed that both Tat-Rac1L61F37A and Tat-Rac1L61Y40C double mutants, each one capable of modulating distinct arms of the Rac1-dependent signaling cascade, were able to promote survival and to prevent dendrite atrophy until 15 days post lesion. In particular, the L61F37A mutant was the most effective and showed a dose-dependent increase of RGC survival. The DN isoform of Rac1 induced opposite effects both on survival and on dendrite morphology, suggesting that the observed effects are not due to the blocking of the RhoGTPase cycling, as indicated in some studies [25], but are rather a specific effect of Rac1 activity modulation. Moreover, we found that the L61F37A mutant was able to promote regeneration until 15 days post-injury and that sustained delivery into the eye by repetitive ivit injections was able to prevent the normal axonal degeneration observed in control nerves at 30 days after injury.

Finally, by immunostaining we observed ERK1/2 upregulation and increase of Pak1 T212 phosphorylation in RGCs after L61F37A mutant treatment. On the other hand after L61Y40C administration we observed an increase of survival, and contextually an ERK1/2 activation specifically in glial cells, raising the possibility that the two Rac1 selective mutants act by different mechanisms. Overall, our study shows that Rac1 is a complex modulator of neuronal survival, morphology and regeneration *in vivo*.

### Activation of Rac1 Downstream Interactors Protected RGCs from Injury-induced Death

To our knowledge, our study is the first demonstrating a direct correlation between Rac1 activation and improvement of neuronal survival *in vivo*. To date, only *in vitro* evidence [9], [10] has been provided, with *in vivo* data showing indirect and controversial findings. For example, one study reported that Rac1 activates the pro-death mixed lineage kinase 3 (MLK3)-JNK kinase signaling pathway promoting neuronal cell death following cerebral ischemia [26], while in another study, Rac1 deletion from germinative zones determined microcephaly [27].

The two mutants used in this work were characterized for the first time in fibroblasts in 1996 by Lamarche and coworkers [13]. In these constitutively active mutants, the effector domain of Rac1 is mutated in order to limit the number of putative downstream interactions. It was shown that the L61F37A mutant retained the ability to activate Pak and indirectly JNK, whereas the L61Y40C mutant displayed opposite effects, blocking the activation of both proteins. However the type and number of downstream interactions are strictly related to the specific cell context and to the model.

Some pathways downstream to Rac1 were found to be neuroprotective *in vitro*: a Rac1-dependent Pak/MEK/ERK1/2 signaling was found to be protective against apoptosis in cerebellar granule neurons [28], [29], and recent studies reported that some neuroprotective agents act by increasing ERK1/2 phosphorylation in the retina [30]–[32]. Moreover, in RGCs the tonic activation of ERK1/2 by overexpression of a constitutively active mitogen-activated protein kinase kinase 1 (MEK1) mutant was able to improve survival after optic nerve injury [33].

In our model we observed by immunostaining an increase in Pak1 T212 phosphorylation in RGCs following L61F37A treatment. This phosphorylation is specifically targeted by the p35/Cdk5 association, whose role in neuroprotection was demonstrated during development and under stress conditions [34]–[36]. Moreover Pak1 phosphorylation by Cdk5 was found to be of critical importance in the complex mechanisms that regulate axonal growth and cell reaction to toxic stimuli [14], [37].

Rac1L61 CA mutant is able to associate to both p35 and Cdk5, while the F37A mutated Rac1 cannot directly bind the p35, indicating that Cdk5 might not be a direct downstream effector of the F37A [14]. Other pathways are likely involved. For example it has been reported many times that ERK stimulates p35 transcription [34] and we found ERK upregulation in RGCs after F37A treatment. A more thorough investigation of the pathways downstream F37A treatment are required and will be object of further studies possibly on more simple experimental models.

Following L61Y40C treatment we did not find any Pak1 or JNK significant alteration, suggesting that the two mutants act by means of different mechanisms. The L61Y40C mutant determined a surprising increase of ERK1/2 activation in radial glia. This result is not easily interpretable since we showed by immunostaining that the Tat-proteins are mainly localized in RGCs. However it must be considered that the Tat-trojan peptide sequence does not act in a cell-selective manner and there is no reason to assume that the Tat-proteins are internalized by the RGCs only, especially in consideration of the close proximity of glial cells to RGCs. Another hypothesis should be that ERK activation in glial cells might be an indirect effect due to a neuron-glia cross-talk. This aspect needs to be investigated in further studies on a more suitable model such as mixed neuron-glia *in vitro* cultures.

The improvement of survival obtained by treatment with the L61Y40C mutant might be related to ERK1/2 activation in glial cells. In retinal astrocytes it was shown that the stimulation of ERK1/2 leads to secretion of endogenous CNTF [38]. It will be interesting to verify, in further studies, the involvement of neurotrophin secretion as intermediate of the Rac1L61Y40C mutant action on neuronal survival.

Inflammation has a well known role in RGC survival [20], [39], but it can be excluded that the findings of our study are related to inflammation processes because the results obtained in control treated mice, by using both the vehicle alone and a Rac1 WT isoform are significantly different from those obtained with the same protocols on experimental mice.

In our experiments at 30 dpl the survival level was the same in control and in treated eyes, and this temporal limitation of action has been observed also for many other agents [40]. Indeed recent studies have shown that the manipulation of a single intracellular pathway is not sufficient to block the apoptotic process for a long period [41].

Rac1 is also a complex regulator of dendrite morphology [42], [43] and this was confirmed by our data, showing that Rac1 activation could prevent the dendrite atrophy normally occurring after injury. In our optic nerve injury model we found, as expected, that selective CA and DN isoforms exerted opposite effects both on neuronal survival and on dendrite morphology, confirming their value as tool to investigate complex phenomena.

### Rac1 Selective Activation Promoted Axonal Regeneration

After optic nerve crush and L61F37A treatment, we found many axons spanning the crush site and run distally in the optic nerve stump, suggesting that this mutant was able to allow the axonal growth through non-permissive environment and to stimulate the intrinsic regenerative program of neurons.

Our findings are in keeping with the study of Jain and coworkers, in which a CA Rac1 was able to promote axonal regeneration in a model of spinal cord injury [8]. Indeed the pathways activated by our L61F37A mutant and the CA Rac1 used by Jain are likely to be partially overlapping. However, Kusano and coworkers obtained the same results by using a DN Rac1 isoform on a model of sciatic nerve injury [11]. The differences might be related to the different context: peripheral versus central nervous system.

The L61F37A ability to increase regeneration could be related to Pak1 T212 phosphorylation. To date data supporting a role for Pak1 in CNS regeneration *in vivo* are missing. Pak modulation in neuroblastoma cells *in vitro* was found to stimulate axonal growth through non-permissive environment and in particular its dead-kinase isoform was found to be very efficient to overcome inhibition [44]. Moreover Pak1 inhibition by the p35/Cdk5 association seems to be important for neurite outgrowth in vitro and for the maintenance of proper neuronal morphology [14], [45]–[47]. It is widely accepted that Pak is a regulator of actin dynamics in many cell types because it regulates LIM kinase (LIMK) and cofilin phosphorylation [48], even if the precise mechanisms are not yet understood. Some studies show that the modulation of Pak1 by p35/Cdk5 and Rac1, could create a signaling complex mechanism aimed at temporally control the activation of downstream events regulating the LIMK- actin/cofilin dynamics [37], [45].

A similar extent of regeneration that we observed in our study after Rac1 selective activation was found also in studies where RhoA was inactivated in RGCs by C3 transferase treatment [18]. RhoA and Rac1 often display opposite effects in many aspects of cell biology [6], and we cannot exclude that the mutants used in this study could indirectly lead to RhoA inactivation. It will be interesting to verify this hypothesis in further experiments.

The extent of regeneration we obtained is small, but we manipulated only a single protein, while it is known that in order to obtain a massive fast axonal growth multiple agents must be administered and many proteins should be manipulated at the same time [41]. The advantage of using the cell penetrating proteins is that they display a pharmacological profile as shown by the dose-dependent effect of Rac1L61F37A mutant on neuronal survival.

### Rac1 Selective Activation Prevented Axonal Degeneration

Another finding of our study is that long-term treatments with the Rac1L61F37A were able to delay the progressive axonal degeneration as compared to undertreated control neurons. To our knowledge, very few, mainly in vitro studies imply Rac1 disregulation in axonal degeneration [49], [50]. One *in vivo* study concerns the modifier of cell adhesion (MOCA), a presenilin binding protein capable of activating Rac1. The loss of MOCA in mice leads to axonal degeneration and causes sensorimotor impairments by decreasing cofilin phosphorylation and altering its upstream signaling partners LIMK and Pak [27]. Although these studies are in accordance with our observations, the possibility of using Rac1 selective activation in order to prevent degeneration should be thoroughly verified in further experiments, for instance by improving the way of administration of the Tat-proteins.

### Conclusions

Our study demonstrates the role of Rac1 in the modulation of neuronal survival, dendrite morphology and axon remodeling and suggests the importance of better defining the intracellular signaling networks triggered by Rac1 small GTPase in neurons. Moreover, it suggests that the modulation of selective Rac1-dependent signaling pathways could be an effective strategy to improve neuron survival, axonal regeneration or to prevent degeneration in the CNS. Recent studies addressing issues concerning the administration of Trojan fusion proteins directly into the brain [51], [52], suggest the realistic possibility of using our Trojan fusion Rac1 mutants as a potential therapeutic approach to modulate neuron survival and axonal regeneration in neurodegenerative diseases.

## Supporting Information

Figure S1 **The Brainbow 1.0 mouse as a tool to study axonal regeneration of optic nerve after injury.** In the Brainbow 1.0 mouse retina only the RFP signal is normally present. After intravitreal injection of AAV-Cre-GFP, a genetic recombination occurs within a few days, allowing the combinatorial expression of YFP and CFP. The RFP is concomitantly deleted during the recombination. Given that the CFP signal was negligible in the optic nerve we decided to show only the YFP. A scheme of the experiment is shown in the upper panel: 2 days after the AAV-Cre-GFP injection a crush lesion was performed. Rac1 mutants or vehicle were ivit injected. During the 15 days post-injection, the recombination occurs only in surviving neurons. Given that recombination occurs after axonal disconnection from the target, the YFP signal will be found only in surviving neurons and in newly formed axonal sprouts. This model allows a clear distinction of the regenerating neurons from the old RFP positive axonal remnants undergoing Wallerian degeneration. Examples of the optic disc and of the optic nerve at the end of the experiment are shown in the lower panels. Here a Rac1L61F37A treated nerve is shown because in control nerves no regenerating axons are present. Given that about the 75% of neurons recombine, the regeneration is underestimated, but this model allows to analyze the whole mounted nerve without need of staining or complex handling. Moreover the whole mounted nerve can be directly acquired by laser scanning microscopy and regeneration can be assessed directly on the confocal stacks.
(TIF)

Figure S2 **Scheme of the Tat-proteins and amino acid sequence.** A) Scheme of the recombinant protein mutants in which all the mutations are indicated: an Histidine tail is followed by a Tat trojan sequence and by the Rac1 protein isoform. The Q61L point mutation tonically activates the protein, the N17 inactivate Rac1, whereas the mutations F37A and Y40C block the interactions with some downstream Rac1 effectors. B) Complete amino acid sequence of the different Rac1 isoforms, where the Histidine tag (red), the Tat sequence (green) and the point mutations (yellow) are highlighted.
(TIF)

Figure S3 **Intracellular uptake of Rac1 mutant after intravitreal injection.** Representative confocal images of whole mounted retinas three days after vehicle (A–B) or Tat-Rac1 protein (C–D) injections showing immunoreactivity for the 6-histidine tail of the recombinant protein (Histag, red), the neuronal marker βIII-tubulin (green) and nuclei (DAPI, blue). A diffuse immunoreactivity for Histag confirmed the successful injection and uptake of the Tat-conjugated mutant. Scale bars 50 µm.
(TIF)

Figure S4 **Recombinant proteins are detectable after 2 days from injection in normal retina.** Retina sections were immunostained by using anti-Histag antibodies at different time points after a single ivit injection of Tat-proteins. A clear positivity was found after 2 days (D to F) but the protein was undetectable at 4 days from the injection (G to I). βIII-tubulin staining (blue in the right panels) confirms the neuronal uptake of the Tat-proteins. GFAP (green) level is low and restricted to the upper layer because retinal astrocytes are not activated. Scale bar 30 µm. GCL: granule cell layer.
(TIF)

Figure S5 **Increased positivity for PAK-p and ERK1/2-p after Rac1L61F37A treatment.** Representative images showing retina sections immunostained by antibodies against the pan-specific and the phosphorylated form of Pak, ERK1/2 and JNK. Retinas were dissected and immunostained 3 days after optic nerve crush and ivit treatment with either vehicle, or Rac1WT, or L61F37A, or L61Y40C. Here we highlight some results relative to the F37A treatment, as complementary of the images shown in Fig.6. After F37A treatment we observed an increase of positivity for ERK1/2-p and PAK-p not found neither in vehicle nor in WT treatments. GCL: granule cell layer; IPL: inner plexiform layer; INL inner nuclear layer. Scale bar 30 µm.
(TIF)

Figure S6 **Rac1 selective activation increased ERK1/2-p and PAK1-p in normal retina.** Examples of retina sections 2 days after a single ivit injection of L61Y40C (A–B) or L61F37A (E–F), without optic nerve crush. An untreated retina is shown as control (C–D, G–H). The treatment with L61Y40C is able to increase ERK1/2-p in non-neuronal cells, as shown by the lacking of colocalization with MAP2. Here the GFAP colocalization is not useful because in normal retina the glia is not activated as happens after crush, so the GFAP level is very low and restricted to the surface of the retina (as shown in F, green). The L61F37A is able to increase the positivity of RGCs for PAK-p (E–F) respect to the control (G–H). Scale bar 20 µm. GCL: granule cell layer.
(TIF)
