# Supplementary figures and images for: Rac1 Selective Activation Improves Retina Ganglion Cell Survival and Regeneration
Source: PLoS One. 2013 May 29;8(5):e64350. doi: 10.1371/journal.pone.0064350 (PMC3667179; doi:10.1371/journal.pone.0064350)

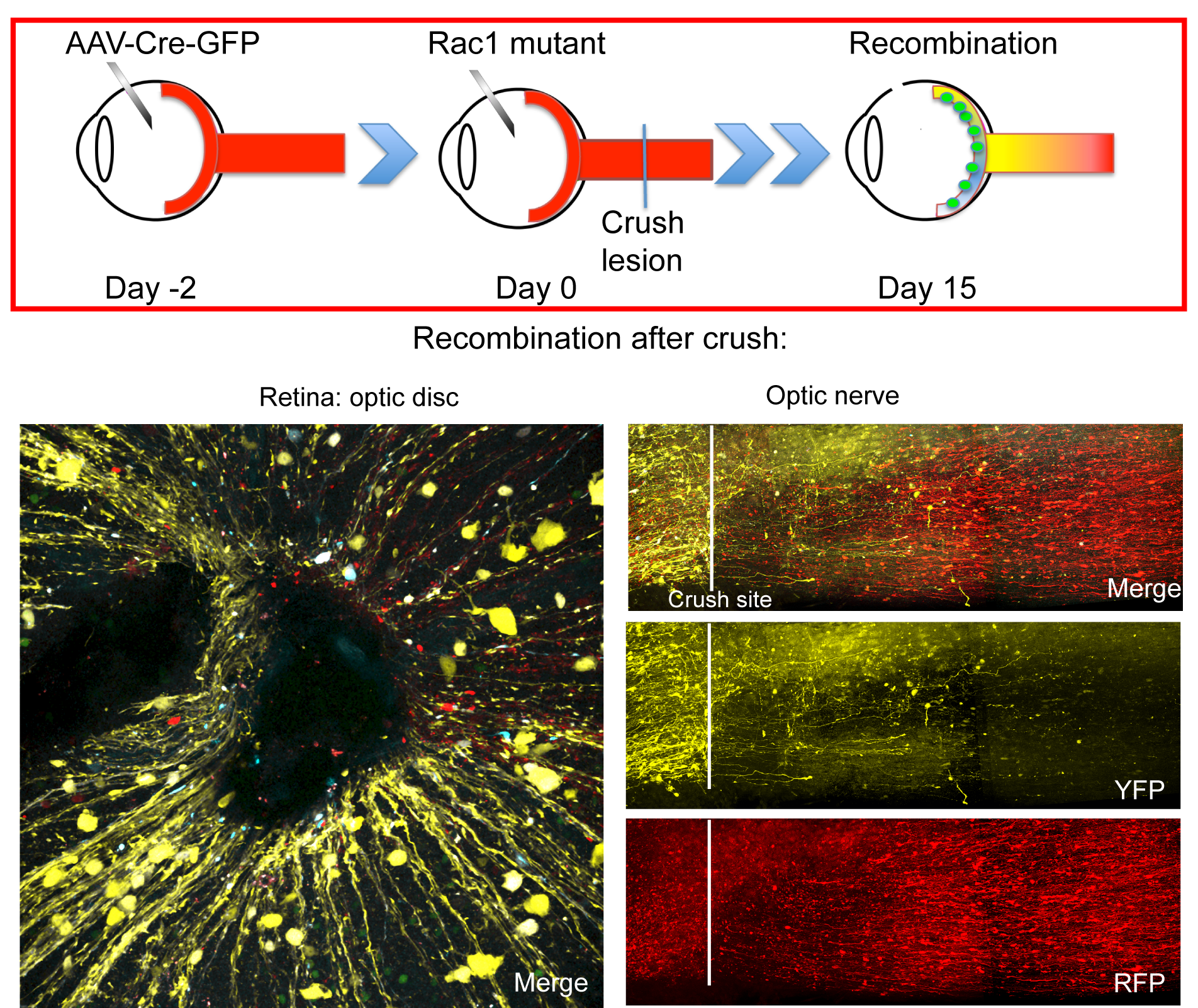

Supplement: Figure S1 — The Brainbow 1.0 mouse as a tool to study axonal regeneration of optic nerve after injury. In the Brainbow 1.0 mouse retina only the RFP signal is normally present. After intravitreal injection of AAV-Cre-GFP, a genetic recombination occurs within a few days, allowing the combinatorial expression of YFP and CFP. The RFP is concomitantly deleted during the recombination. Given that the CFP signal was negligible in the optic nerve we decided to show only the YFP. A scheme of the experiment is shown in the upper panel: 2 days after the AAV-Cre-GFP injection a crush lesion was performed. Rac1 mutants or vehicle were ivit injected. During the 15 days post-injection, the recombination occurs only in surviving neurons. Given that recombination occurs after axonal disconnection from the target, the YFP signal will be found only in surviving neurons and in newly formed axonal sprouts. This model allows a clear distinction of the regenerating neurons from the old RFP positive axonal remnants undergoing Wallerian degeneration. Examples of the optic disc and of the optic nerve at the end of the experiment are shown in the lower panels. Here a Rac1L61F37A treated nerve is shown because in control nerves no regenerating axons are present. Given that about the 75% of neurons recombine, the regeneration is underestimated, but this model allows to analyze the whole mounted nerve without need of staining or complex handling. Moreover the whole mounted nerve can be directly acquired by laser scanning microscopy and regeneration can be assessed directly on the confocal stacks. (TIF) [file pone.0064350.s001.tif]

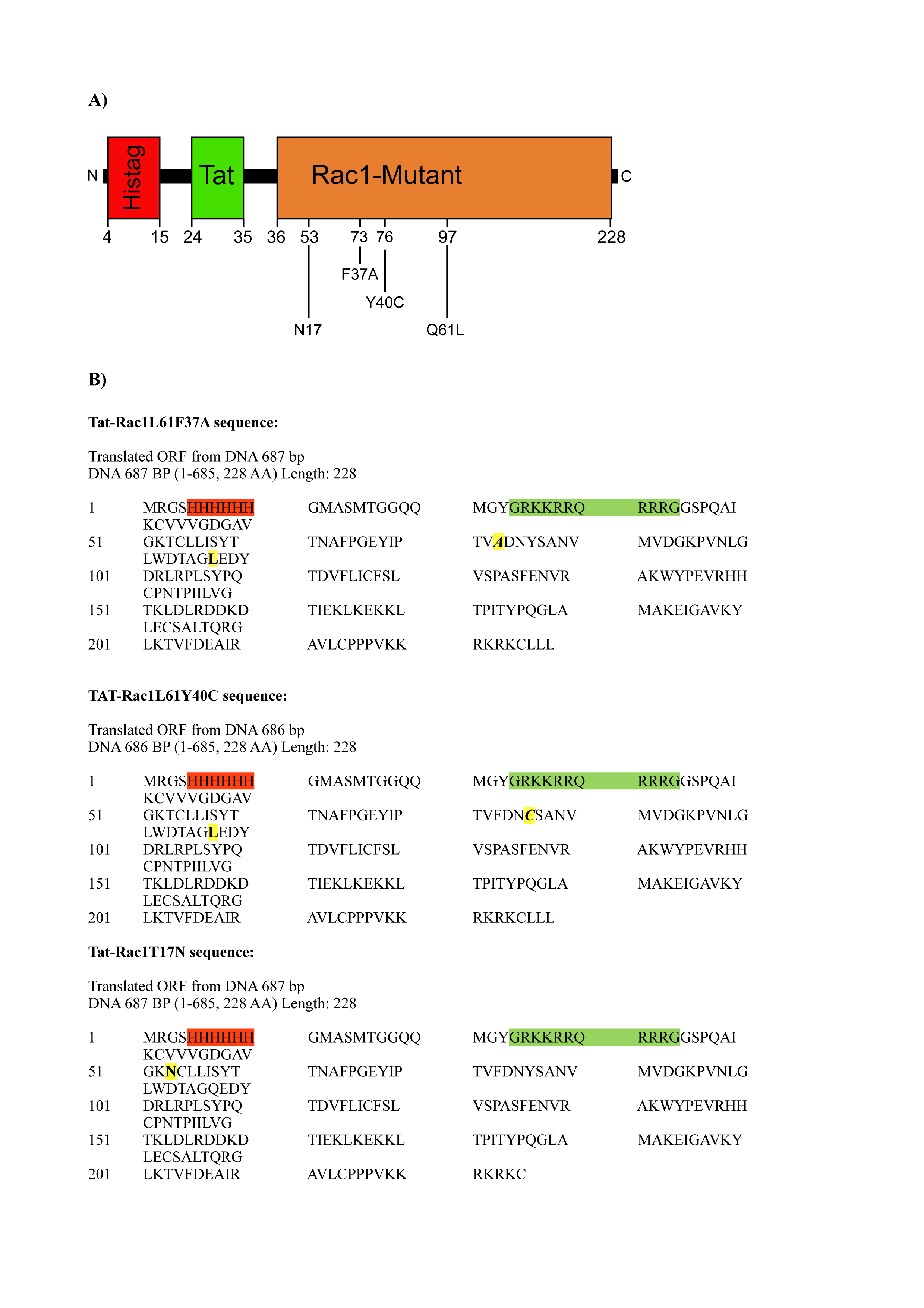

Supplement: Figure S2 — Scheme of the Tat-proteins and amino acid sequence. A) Scheme of the recombinant protein mutants in which all the mutations are indicated: an Histidine tail is followed by a Tat trojan sequence and by the Rac1 protein isoform. The Q61L point mutation tonically activates the protein, the N17 inactivate Rac1, whereas the mutations F37A and Y40C block the interactions with some downstream Rac1 effectors. B) Complete amino acid sequence of the different Rac1 isoforms, where the Histidine tag (red), the Tat sequence (green) and the point mutations (yellow) are highlighted. (TIF) [file pone.0064350.s002.tif]

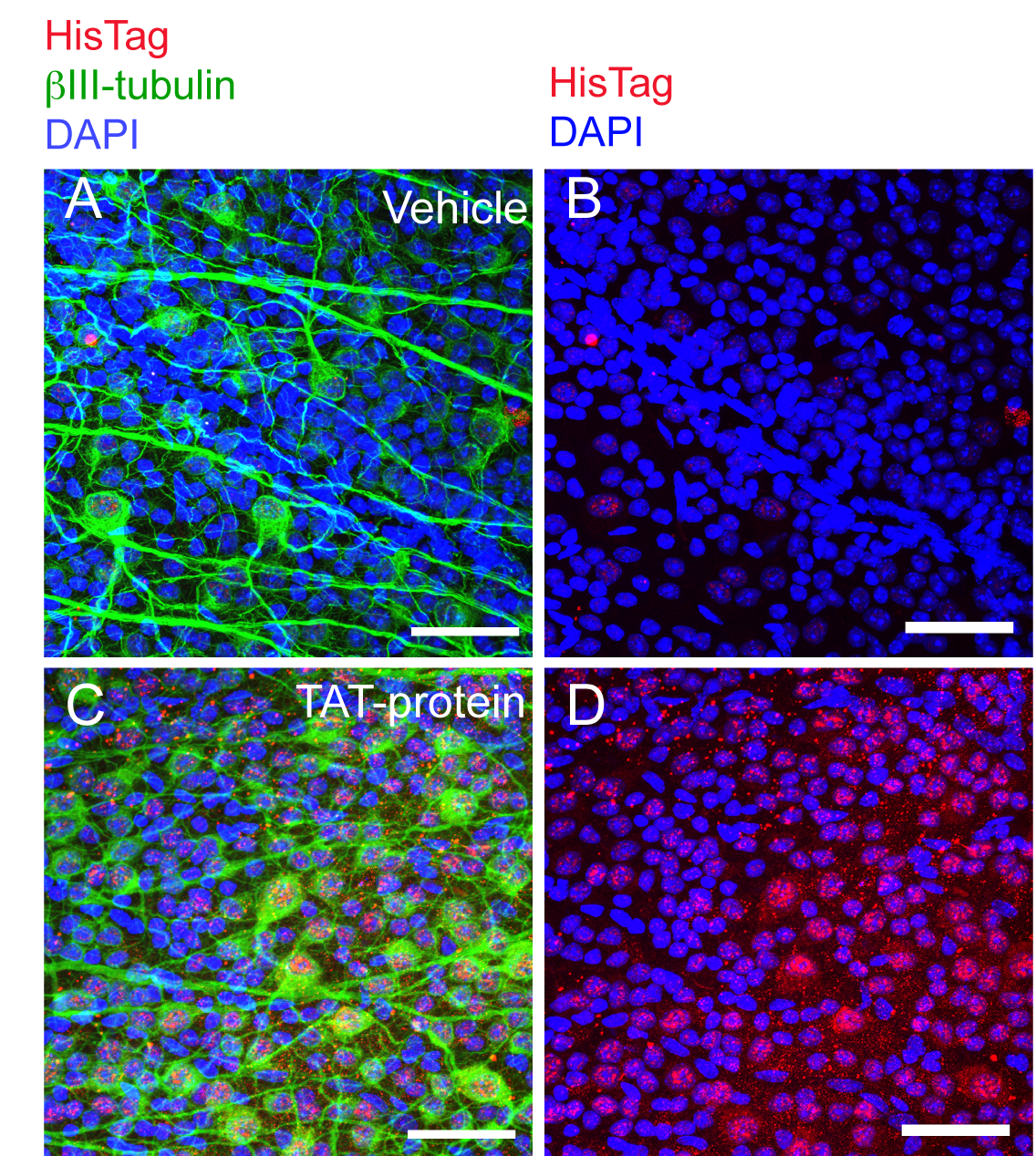

Supplement: Figure S3 — Intracellular uptake of Rac1 mutant after intravitreal injection. Representative confocal images of whole mounted retinas three days after vehicle (A–B) or Tat-Rac1 protein (C–D) injections showing immunoreactivity for the 6-histidine tail of the recombinant protein (Histag, red), the neuronal marker βIII-tubulin (green) and nuclei (DAPI, blue). A diffuse immunoreactivity for Histag confirmed the successful injection and uptake of the Tat-conjugated mutant. Scale bars 50 µm. (TIF) [file pone.0064350.s003.tif]

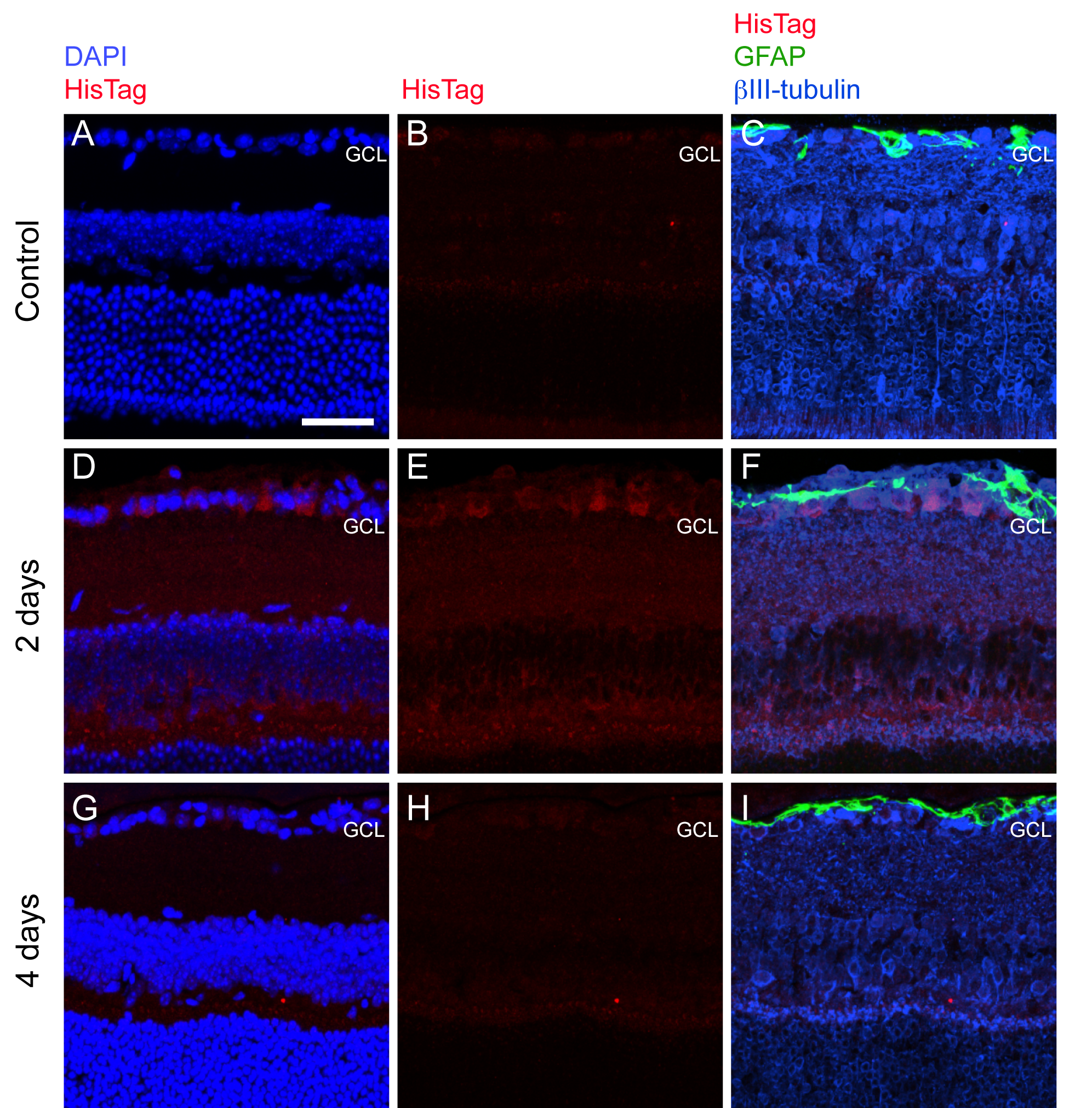

Supplement: Figure S4 — Recombinant proteins are detectable after 2 days from injection in normal retina. Retina sections were immunostained by using anti-Histag antibodies at different time points after a single ivit injection of Tat-proteins. A clear positivity was found after 2 days (D to F) but the protein was undetectable at 4 days from the injection (G to I). βIII-tubulin staining (blue in the right panels) confirms the neuronal uptake of the Tat-proteins. GFAP (green) level is low and restricted to the upper layer because retinal astrocytes are not activated. Scale bar 30 µm. GCL: granule cell layer. (TIF) [file pone.0064350.s004.tif]

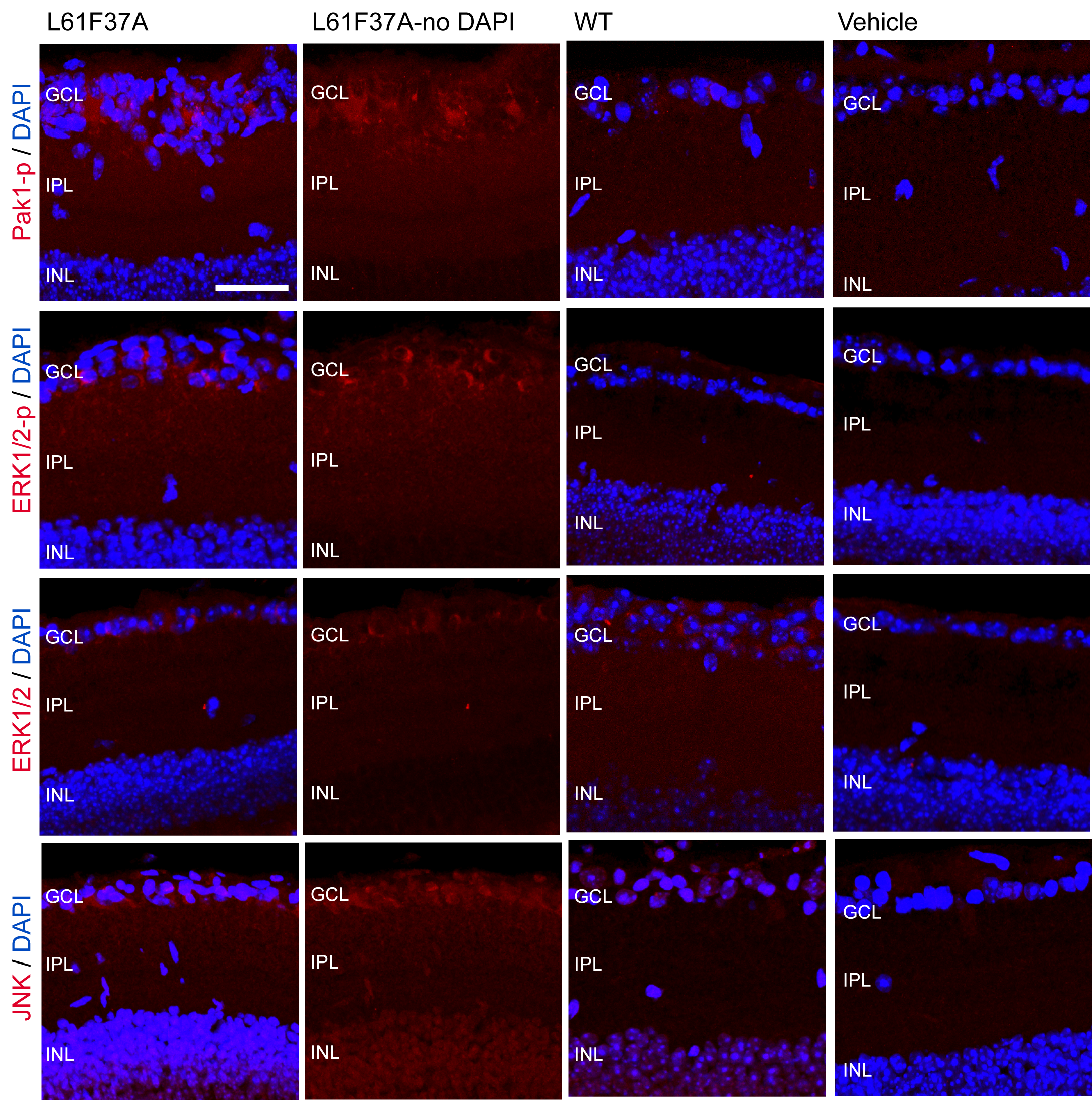

Supplement: Figure S5 — Increased positivity for PAK-p and ERK1/2-p after Rac1L61F37A treatment. Representative images showing retina sections immunostained by antibodies against the pan-specific and the phosphorylated form of Pak, ERK1/2 and JNK. Retinas were dissected and immunostained 3 days after optic nerve crush and ivit treatment with either vehicle, or Rac1WT, or L61F37A, or L61Y40C. Here we highlight some results relative to the F37A treatment, as complementary of the images shown in Fig.6. After F37A treatment we observed an increase of positivity for ERK1/2-p and PAK-p not found neither in vehicle nor in WT treatments. GCL: granule cell layer; IPL: inner plexiform layer; INL inner nuclear layer. Scale bar 30 µm. (TIF) [file pone.0064350.s005.tif]

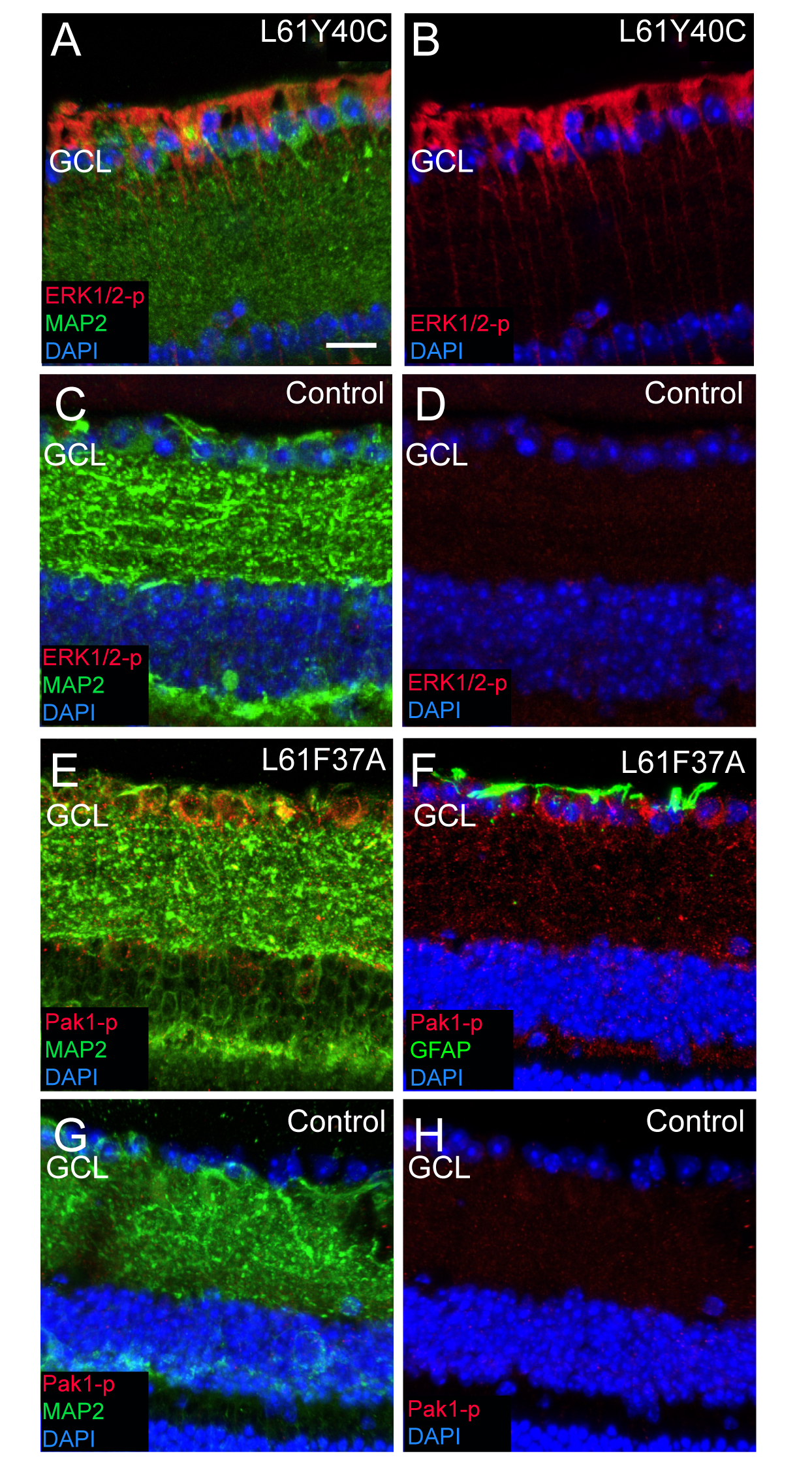

Supplement: Figure S6 — Rac1 selective activation increased ERK1/2-p and PAK1-p in normal retina. Examples of retina sections 2 days after a single ivit injection of L61Y40C (A–B) or L61F37A (E–F), without optic nerve crush. An untreated retina is shown as control (C–D, G–H). The treatment with L61Y40C is able to increase ERK1/2-p in non-neuronal cells, as shown by the lacking of colocalization with MAP2. Here the GFAP colocalization is not useful because in normal retina the glia is not activated as happens after crush, so the GFAP level is very low and restricted to the surface of the retina (as shown in F, green). The L61F37A is able to increase the positivity of RGCs for PAK-p (E–F) respect to the control (G–H). Scale bar 20 µm. GCL: granule cell layer. (TIF) [file pone.0064350.s006.tif]
